# Bacterial vaginosis and specific high-risk human papillomavirus genotype infection: a cross-sectional study in Nairobi, Kenya

**DOI:** 10.3389/fgwh.2026.1816885

**Published:** 2026-06-18

**Authors:** Raymond Chibvongodze, Mutinda Cleophas Kyama, Lucy Muchiri

**Affiliations:** 1 Department of Human Pathology, Faculty of Health Sciences, University of Nairobi, Nairobi, Kenya; 2 Department of Medical Laboratory Sciences, Jomo Kenyatta University of Agriculture and Technology, Nairobi, Kenya

**Keywords:** Africa, bacterial vaginosis, cervical cancer, epidemiology, human papillomavirus, odds ratio, pap smear, polymerase chain reaction

## Abstract

**Background:**

Emerging evidence indicates that bacterial vaginosis (BV) plays a role in high-risk Human Papillomavirus (hr-HPV) infection. However, the BV/hr-HPV association is still an under-explored concept in Africa, a region overburdened by the highest prevalence of both infections and cervical cancer in the world. Additionally, there is a significant knowledge gap regarding which specific hr-HPV genotypes contribute to this association in Africa. The objective of this study was to investigate whether BV is significantly associated with hr-HPV infection in Africa, with particular emphasis on the specific hr-HPV genotypes contributing to this association.

**Methods:**

This publication is based on a cross-sectional analysis of baseline data from an ongoing cohort study. A total of 498 women met the eligibility criteria and were screened for BV, hr-HPV, and cervical lesions at baseline between July 2022 and November 2023. Logistic regression, C*hi*-square tests, and *t* tests were used to investigate associations among hr-HPV genotypes, BV, cytology results, and other risk factors. In all tests, a *p*-value of <0.05 was considered to indicate statistical significance.

**Results:**

The BV infection and hr-HPV infection rates were 24.7% (123/498) and 28.1% (140/498), respectively. Almost one-tenth (49/498) of the enrolled women had BV/hr-HPV coinfection. A significant association between BV and pooled hr-HPV status (inclusive of the HPV-16, -18, -31, -33, -35, -39, -45, -51, -52, -56, -58, -59, -66, and -68 genotypes) was confirmed in this study (aOR = 2.38, 95% CI: 1.48–3.81), *p* < 0.001). BV was significantly associated with HPV-39 and HPV-52 genotypes (aOR: 3.23, 95% CI: 1.37–7.64, *p* = 0.007) and (aOR: 5.38, 95% CI: 1.17–24.74; *p* = 0.03), respectively. HPV-16 and HPV-68 were significantly associated with abnormal Pap smears (*p* = 0.046 and *p* = 0.001, respectively).

**Conclusions:**

BV was found to be significantly associated with pooled hr-HPV status, HPV-39, and HPV-52 infection in Kenyan women. These findings underscore the importance of treating BV promptly, as doing so may help minimize hr-HPV infection.

## 1 Introduction

Cervical cancer is the fourth most common malignancy among women worldwide (1). In 2022, approximately 570,000 women were diagnosed with cervical cancer worldwide, and almost 310,000 died from it in the same year (1). However, in Kenya, cervical cancer is the second most common malignancy after breast cancer (2). In 2020, approximately 5,236 women were diagnosed with cervical cancer in Kenya, and 3,211 died from it (2). The high prevalence of cervical cancer in Africa is due to very low HPV vaccine coverage in young women, poor cervical cancer screening awareness, and public cervical cancer screening programs that are limited by poor coverage and accessibility (3).

HPV infection is the most common sexually transmitted infection, and over 80% of women are infected by the virus by the age of 45 years (4). In 2020, the prevalence of HPV was reported to be 23.6% in Kenya (1). However, most of these HPV infections (>90%) are transient and spontaneously resolve within 24 months; only a minority of these HPV infections persist (4). Persistent HPV infection is a prerequisite for the development of cervical intraepithelial neoplasia (CIN) (5). There are >100 HPV genotypes, and only 14 are considered high-risk (hr) genotypes, including 16, 18, 31, 33, 35, 39, 45, 51, 52, 56, 58, 59, 66, and 68 (6, 7).

Bacterial vaginosis is a common polymicrobial vaginal disorder characterized by depletion of *Lactobacillus* species, followed by an overgrowth of anaerobic bacteria such as *Gardnerella vaginalis, Prevotella, Mobiluncus, Bacteroides species, and Mycoplasma hominis* (8). Patients with BV present with foul-smelling vaginal discharge, vaginal itchiness, a burning sensation during urination, and increased vaginal pH levels (9). However, a significant proportion of patients remain asymptomatic (10). The prevalence of bacterial vaginosis was reported to be 23.0% in Kenyan women by Owono et al. (11). BV can be diagnosed via Amsel's criteria, identification of clue cells on Papanicolaou-stained vaginal smears, the Nugent criteria, and BV polymerase chain reaction (PCR) (8), all of which are subjective except for BV PCR.

Emerging evidence indicates that BV plays a role in HPV infection (12). However, the BV/hr-HPV association is still an under-explored concept in Africa, a region overburdened by the highest prevalence of both infections and cervical cancer in the world. Additionally, there is a significant knowledge gap as to which specific hr-HPV genotypes contribute to this association in Africa. The association between BV and specific hr-HPV genotypes remains underexplored, and few studies have examined this relationship. In a study by Menon et al.*,* a borderline association between BV and HPV-58 (*p* = 0.07) was reported as an incidental finding (7). Lin et al. reported that BV was significantly associated with HPV-51 and -52 (12). However, Lin et al. acknowledged that the utility of Amsel's criteria for detecting BV in their study may have introduced subjectivity and high inter-observer variability (12). This limitation was overcome in this study by using the polymerase chain reaction (PCR), which is more objective.

BV is postulated to promote HPV infection and persistence through several mechanisms (10). Anaerobes associated with BV produce sialidases, which break down mucus, thereby exposing the epithelium to infection by several microorganisms, including HPV (10). Borgdorff et al. reported that BV destabilizes the epithelial cytoskeleton, leading to epithelial exfoliation and necrosis, thereby facilitating HPV infection (13). BV induces the production of chemical mediators of inflammation, such as lipoteichoic acid, lipopolysaccharides, and peptidoglycans, that facilitate *E*6 and *E*7 expression, which aids the integration of the HPV genome into the host genome (10). According to Straight et al.*,* the acidic pH mediated by *Lactobacillus*-dominated vaginal microbiota is protective against HPV infection and persistence, as the HPV early gene protein (*E*5) is susceptible to acidic pH (14). However, reverse causality between BV and HPV infection cannot be excluded. This is because it is postulated that HPV infection alters the vaginal microbiota through elicitation of the host mucosal immune response and initiation of genital inflammation (15).

The objective of this study was to investigate whether BV is significantly associated with hr-HPV infection in Africa, with particular emphasis on the specific hr-HPV genotypes contributing to this association. This work is part of an ongoing cohort study (reference number: P107/02/2019) that aims to investigate whether BV is associated with a higher risk of hr-HPV persistence.

## 2 Materials and methods

### 2.1 Study design

This was a cross-sectional study with a broad objective to investigate whether BV is significantly associated with hr-HPV infection in Africa, with particular emphasis on determining the specific hr-HPV genotypes contributing to this association. The study period was between July 2022 and November 2023.

### 2.2 Study setting

The study was conducted at Kenyatta National Hospital (KNH) Clinic 66 (Reproductive Health Clinic), KAVI Institute of Clinical Research (KAVI ICR), KNH Cytology Laboratory, and Kenya Medical Research Institute (KEMRI).

### 2.3 Study population and eligibility for enrollment

Women attending Clinic 66 at KNH for cervical cancer screening services were recruited in this study. The inclusion criterion was a non-pregnant woman aged ≥30 years with no recent treatment for bacterial vaginosis (six months). The exclusion criteria in this study were a prior history of cervical cancer, CIN2/3 or carcinoma in situ (CIS) histopathology results, or high-grade squamous intraepithelial (HSIL) cytopathology results. Women under 30 years of age were excluded from this study because transient infections, which are self-limiting, are very common in this age group. According to the American College of Obstetricians and Gynecologists, cotesting women younger than 30 years results in the detection of transient infections and would result in more frequent and unnecessary follow-up testing (16).

### 2.4 Data collection and sample collection

Four nurses from Clinic 66 were recruited as research assistants to assist with data collection. The quantitative data were gathered through face-to-face interviews using a structured questionnaire with closed-ended questions. Data gathered at the baseline assessment included demographic variables (age), reproductive health variables (vaginal douching practice, age of sexual debut, HIV status, number of sexual partners over the past two years, parity, and last menstrual period), and lifestyle variables (history of smoking and history of alcohol consumption).

After the patients filled and signed the consent forms, they were placed in a dorso-lithotomy position on the examination bed, and an unlubricated speculum was inserted into the vagina. First, a sterile cotton swab was rubbed against the vaginal lateral wall to collect a vaginal sample, which was immediately preserved in Universal Transport Medium (Copan, Brescia, Italy). This sample was used for PCR for both BV and hr-HPV. Next, a cervical sample was collected using a Cervex brush (Rovers, Oss, The Netherlands), smeared onto a microscope slide, and fixed with 95% alcohol. The samples were labeled with unique study numbers and immediately dispatched to the laboratory for processing. The samples were either processed immediately (Pap smears) or stored at −80°C prior to processing (PCR samples for BV and PCR). To standardize sample collection skills, a half-day training session was held at the clinic in July 2022.

### 2.5 Laboratory processes

#### 2.5.1 PCR detection of hr-HPV

hr-HPV DNA testing was performed via the commercial Sacace HPV Genotypes 14 Real-TM Quant test kit (CE-IVD) (Sacace Biotechnologies, Como, Italy), which detects fourteen high-risk genotypes: 16, 18, 31, 33, 35, 39, 45, 51, 52, 56, 58, 59, 66, and 68. HPV DNA was extracted from the vaginal swabs via the QIAamp DNA Mini Kit (Qiagen, Hilden, Germany) according to the manufacturer's instructions, and a 100 µL aliquot was produced. The final PCR volume (25 µL) was produced by mixing 10 µL of the HPV DNA sample and 15 µL of the reaction mixture. The qualitative PCR assays were performed using a Rotor-Gene Q (Qiagen, Hilden, Germany). The thermal cycles applied sequentially were 95°C for 15 min; 5 cycles at 95°C for 5 s, 60°C for 20 s, and 72°C for 15 s; and 40 cycles at 95°C for 5 s, 60°C for 30 s, and 72°C for 15 s. The fluorescence generated was read in the FAM, Hex, ROX, and Cy5 channels.

To ensure quality during the analysis phase, an internal control (*human beta-globin gene*) provided in the kit was used. The *human beta-globin gene* encodes proteins involved in basic cellular functions and is therefore used to assess the quality of clinical swabs, namely, whether enough cells are collected. Cellularity of the clinical samples was assessed by quantitative PCR (qPCR) of the *human beta-globin gene* in human cells, using the Cy5 fluorescence channel. Since HPV is an intracellular organism, it is important to demonstrate that a sample that tested HPV-positive also contained sufficient cells. Therefore, qPCR of the *human beta-globin gene* in human cells is essential to confirm that the HPV detected in the sample originated from the sample. Conversely, positive *human beta-globin gene* amplification (internal control) and a negative HPV result confirmed that the clinical sample lacked HPV and was not due to non-amplification.

hr-HPV testing was not routinely being conducted at the hospital when this study was conducted. The researchers offered hr-HPV testing to women who had already reported to Clinic 66 for Pap smear services. Therefore, parallel cotesting for hr-HPV and Pap smear cytology was performed in this study. hr-HPV testing was not conducted as a reflex test in this study.

#### 2.5.2 PCR detection of bacterial vaginosis

BV detection was carried out via the commercial Vircell Vaginal Panel Real-time PCR Kit (CE-IVD) (Vircell Molecular, Granada, Spain), which detects BV on the basis of the relative quantities of three markers: *Lactobacillus* spp.*, Gardnerella Vaginalis, and Atobium Vaginae*. Bacterial DNA was extracted from vaginal swabs using the QIAamp DNA Microbiome Kit (Qiagen, Crawley, United Kingdom). A 100 μl elution mixture was prepared, and 5 μl was added to a “mix A” PCR tube (for detection of *Lactobacillus* spp.*, G. Vaginalis, and A. Vaginae).* The quantitative PCR (qPCR) assays were carried out using a Bio-Rad (CFX96 TouchTM) RT‒PCR Detection System Thermal Cycler. (Bio-Rad Laboratories, Hercules, USA). The targets for detection were ***the 16S gene, rplK gene, and 16S gene*** for *G. Vaginalis, Lactobacillus* spp., *and A. Vaginae,* respectively. VIRCOM molecular communication software automatically calculates the relative amounts of the three targets (*G. vaginalis*, *Lactobacillus* spp., *and A. Vaginae*) and classifies the vaginal microbiota into four grades (G1-G4). G1 and G2 were classified as normal flora, whereas G3 and G4 were classified as bacterial vaginosis.

The vaginal microbiota was classified as G1 if only *Lactobacillus* spp. were detected. The vaginal microbiota was classified as G2 if it was dominated by *Lactobacillus* spp. but contained few *G. vaginalis* and/or *A. vaginae.* The vaginal microbiota was classified as G3 if it was dominated by *G. vaginalis/A. vaginae,* but also contained *Lactobacillus* spp. The vaginal microbiota was classified as G4 in two situations. The first was when the vaginal flora was dominated by *G. vaginalis/A. vaginae* with a paucity or absence of *Lactobacilli* spp. The second situation occurred when *Lactobacilli* spp. were scarce or absent in a sample, and no detection of the three target bacteria was observed. Failure to detect the three target bacteria may be due to BV caused by other bacteria not covered by the kit, or to antibiotic use before vaginal sample collection. The amplification process and sample integrity were assessed by analyzing the negative and positive controls provided with the qPCR kit.

The Vircell Vaginal Panel Real-time PCR kit was imported from Europe for this research only. The researchers relied on clinical validity data as reported by Amor et al.*,* who evaluated the performance of this Vircell Vaginal Panel Real-time PCR Kit in Spain and yielded a sensitivity, specificity, positive predictive value, and negative predictive value of 93.1%, 88.8%, 90.1% and 92.2%, respectively (18). BV PCR results were not used to make clinical decisions because the kit was not validated for clinical purposes within the hospital. Patients primarily came for cervical cancer screening, and therefore, Pap smear and hr-HPV PCR results guided clinicians to make clinical decisions.

#### 2.5.3 Pap smear processing and interpretation

Pap smears were stained with Papanicolaou stain and evaluated by two independent primary individuals, a cytologist and a pathologist. Discrepant findings were referred to a third person, a pathologist with extensive experience in cytopathology. The 2014 Bethesda System for reporting cervical cytology was used to report the cytology findings (19). Patients with abnormalities were referred to a gynecologist within the same clinic for further management. To ensure the reliability of laboratory results, all procedures were performed in accordance with the respective SOPs.

### 2.6 Statistical analyses

#### 2.6.1 Sample size determination

The sample size calculation was determined using the Fisher's formula as follows:-

$$n=\;\frac{Z_{\alpha }^{2}\;\times \;p\;\times \;(1-p)}{d^{2}}$$

Where:

- *n* *=* required sample size
- *Z* *=* Z-score corresponding to a 95% confidence level (1.96)
- *p* *=* estimated proportion of the population with the attribute of interest. (0.2)
- *d* *=* margin of error, set at 5% (0.05).

Data from a study by Dahoud et al. (17) were used to calculate the sample size. In Dahoud's study, the proportion of women who had BV/HPV infections was 20.3%.

Calculation:

$$n=\;\frac{1.96^{2}\;\times \;0.2\;\times \;(1-0.2)}{0.05^{2}}$$

$$n=\;\frac{3.8416\;\times \;0.16}{\;0.0025}\;\;\;\;\approx 249$$

After adjusting for a potential 50% buffer rate, the final target sample size was:

$$n_{adj}=\frac{249}{1-0.5}=\frac{249}{0.5}$$

n=498

Adjusting for a 50% buffer rate yielded a final sample of **498 respondents**, distributed proportionately.

#### 2.6.2 Buffer justification

A larger sample size was used to ensure a higher statistical power, better representation of heterogeneity, increased precision, reduced standard error, narrower confidence intervals, and decreased risk of type II errors (missing true effects). The cohort study (parent study) also required splitting the sample into two cohorts: the (BV-positive/hr-HPV-positive cohort) and the (BV-negative/hr-HPV-positive cohort). Therefore, a larger sample was necessary for the authors to have sufficient numbers in both cohorts to permit valid analysis. Additionally, the population was highly diverse and highly variable; therefore, a larger sample was essential to accurately capture diversity that could have been missed by the minimum sample size without a buffer. The larger sample size was also necessary to account for potential dropouts, non-respondents, or missing data. Moreover, the buffer increased the likelihood of detecting small but meaningful effect sizes, boosting statistical power and reducing bias, thus making the final findings more convincing, reliable, and representative of the population.

#### 2.6.3 Study variables

The variables assessed in this study are summarized in Table 1 below. These variables were captured as per self-report by the patients. Multicollinearity diagnostics were conducted to assess whether any two or more independent variables were highly correlated; such correlations could distort the reliability of the regression coefficients. A variance inflation factor (VIF) >5 indicates severe multicollinearity, while a tolerance <0.10 suggests a serious issue.

**Table 1:** Independent variables and their measurement.

Variables	Measurements
Patient age and age of sexual debut (continuous)	Continous in years
HIV status (nominal)	Captured as negative, unknown (never tested for the past 2 years), or positive.
Parity (nominal)	Dichotomized into “parous” or ‘nalliparous”
Number of sexual partners for the past 2 years (nominal)	Dichotomized into “≤1 person”or “≥2 persons”
Condom use (nominal)	Dichotomized into “always” or “irregular”
Vaginal douching (nominal)	Dichotomized into “yes” for those who practice it and “no” for those who do not.
History of smoking (nominal)	Dichotomized into “yes” for those who ever smoked and “no” for those who had never smoked.
History of alcohol consumption (nominal)	Dichotomized into “yes” for those who ever consumed alcohol and “no” for those who had never consumed alcohol.

#### 2.6.4 Data processing and analysis

The demographic and behavioral qualitative data gathered via a paper-based questionnaire were coded before being entered into Microsoft Excel and then exported to SPSS version 29 for cleaning and analysis. Categorical variables are presented as frequencies and percentages. Continuous variables are reported as the means, standard deviations, medians, and interquartile ranges (IQRs).

Two-sided independent *t* tests were used to compare the patient age and age of sexual debut between patients with BV or hr-HPV with patients without BV or hr-HPV infection. Two-sided independent *t* tests were also used to compare BV or HPV rates between HIV negative and HIV positive patients. Univariate logistic regression analyses were conducted to identify risk factors associated with either hr-HPV infection or BV. A multivariable logistic regression analysis was conducted to assess the adjusted strength of the association between BV and hr-HPV infection, and to control for potential confounders simultaneously. The primary variables for adjustment were age, vaginal douching practice, number of sexual partners, and HIV status because they were independently associated with either BV or hr-HPV. However, to improve the generalizability of the study findings, adjustments were also made for age at sexual debut, condom use, parity, smoking, and alcohol consumption. During the analysis of the association between BV and multiple HPV genotypes, a Benferroni correction was not done to avoid the likelihood of type II errors. Including the Benferroni correction could also have drastically reduced statistical power and made it difficult to detect true associations. The Chi-square test was used to investigate the association between hr-HPV infection and Pap smear findings. In all analyses, a *p* value <0.05 was considered to indicate statistical significance.

### 2.7 Ethical statement

This study was approved by the Kenyatta National Hospital – University of Nairobi Ethics and Research Committee (KNH-UoN ERC), reference number: (P107/02/2019). Written informed consent for participation and the publication of anonymized patient data was obtained from all study participants. This study was not a clinical trial, and the investigators had no control over clinical decisions made after testing. In this study, ethical principles were followed in accordance with the Declaration of Helsinki.

## 3 Results

**Figure 1:**
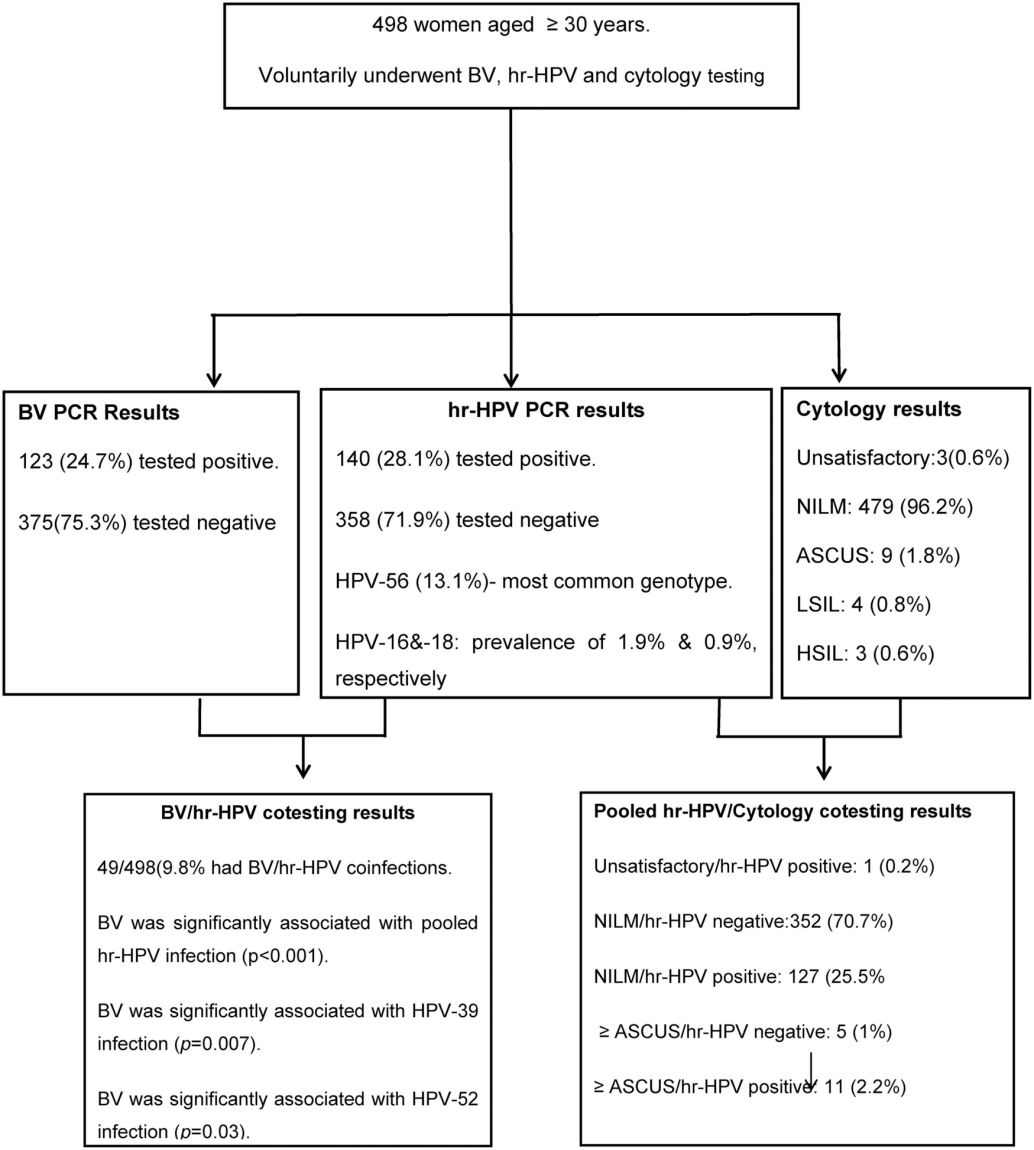
Flow chart of the study protocol.

A total of 498 consenting and non-pregnant women aged ≥30 years met the eligibility criteria for this study. All women were screened for hr-HPV, BV, and cervical lesions (using Pap smears) at baseline. Figure 1 below summarises the findings of the study.

### 3.1 Baseline patient characteristics

The mean (SD) age of the study participants was 40.8 (9.0) years. Of the enrolled women, 56.3% (281/498) were ≤40.8 years old (mean age). Women with BV were older than women without BV (mean ages: 44.8 and 39.4 years, respectively; *p* = 0.01). There was no significant difference in the ages of hr-HPV-infected women and women without hr-HPV infection (mean ages: 39.7 and 41.3 years, respectively; *p* = 0.46). There was no significant difference in the detection rate of abnormal cytology results between women ≤40.8 years (mean age) and women > 40.8 years [3.7% (10/270) vs. 2.9% (6/209), respectively; *p* = 0.38].

The mean (SD) age at sexual debut was 18.8 (2.1) years. There was no significant difference in the mean age at sexual debut between women without BV and women with BV (18.9 vs. 18.7 years, respectively, *p* = 0.55). The mean ages at sexual debut of hr-HPV-negative and hr-HPV-positive women were comparable (18.8 and 19.0 years, respectively, *p* = 0.65).

Most women in this study, 86.7% (432/498), were HIV negative, 10.2% (51/498) were HIV positive, and 3.0% (15/498) were not aware of their HIV status because they had not been tested over the past two years. Compared with their HIV-negative counterparts, HIV-positive women had higher rates of BV [47.0% (24/51) vs. 21% (91/432), respectively, *p* < 0.001]. Therefore, HIV-positive women had greater odds of developing BV than their HIV counterparts did (cOR: 3.33, 95% CI: 1.83–6.95; *p* < 0.001). The prevalence of hr-HPV infection in HIV-negative and HIV-positive women was comparable [28.0% (121/432) vs. 31.0% (16/51), respectively, *p* = 0.31]. The 15 patients who were not aware of their HIV status were excluded from this statistical analysis.

The number of sexual partners over the past two years ranged from 0 to 11. The majority, 85.1% (424/498), had ≤1 partner, and 14.9% (74/498) had ≥2 partners. The median (range) number of parturitions was 2 (0–7). The majority were parous (90.6%; 451/498), and only 9.4% (47/498) were nulliparous. These patient characteristics are summarized in Table 2 below.

**Table 2:** Baseline patient characteristics.

Age		*N* (%)	Mean	SD
≤ 40.8 years (mean age)		281 (56.3%)	40.8	9.0
> 40.8 years		217 (43.5%)		
Age at sexual debut (years)			18.8	2.1
Condom Use	Always	60 (12.0%)		
	No regular use	438 (88.0%)		
Vaginal Douching	Yes	91 (18.3%)		
	No	407 (81.7%)		
No of sexual partners	≤1 partners	424 (85.1%)		
	≥2 partners	74 (14.9%)		
Parity	Parous	451 (90.6%)		
	Naliparous	47 (9.4%)		
HIV status	Negative	432 (86.7%)		
	Positive	15 (3.0%)		
	Unknown	51 (10.2%)		
Smoking	Yes	14 (2.8)		
	No	484 (97.2)		
Alcohol Consumption	Yes	42 (8.4)		
	No	456 (91.6)		

### 3.2 BV prevalence

BV was diagnosed in 24.7% (123/498) of the women (grades 3 and 4). The majority of the women (75.3%, 375/498) had normal vaginal microbiota (grades 1 and 2). The frequencies of each grade are displayed in Table 3 below. The age of the women with BV ranged from 30 to 81 years, with a mean (SD) of 39.5 (8.3) years. Women with BV were older than women without BV (mean ages: 44.8 and 39.4 years, respectively; *p* = 0.01). A significant association between BV and vaginal douching was demonstrated (cOR: 1.66, 95% CI = 1.01–2.72; *p* = 0.04).

**Table 3:** PCR results for BV detection.

Grade	Microbiota Profile	Interpretation	*N* (%)
1	Only *LB* spp. were detected.	Normal flora	310 (62.2)
2	Predominance of *LB* spp, *GV/AV* present.	Normal flora	65 (13.1)
3	Predominance of *GV/AV*, *LB* spp present	BV	81 (16.3)
4	Predominance of *GV/AV* and scarcity of *LB* spp OR scarcity of *LB* without detection *of GV/AV.*	BV	42 (8.4)

LB, *Lactobacillus*; GV, Gardenerella vaginitis; AV, Atobium vaginae.

### 3.3 hr-HPV prevalence

Among the 498 individuals enrolled, 140 (28.1%) tested positive for hr-HPV. Among these hr-HPV-positive samples, 58.6% (*n* = 82/140) had single hr-HPV genotype infections, and a significant proportion (41.4%, *n* = 58/140) had multiple hr-HPV genotype infections. The majority of those with multiple hr-HPV genotypes had dual infections (79.3%, *n* = 46/58); the remaining cases are summarized in Table 4 below. Therefore, the total number of hr-HPV genotypes detected in this study was 211.

**Table 4:** Number of coinfections in patients with multiple hr-HPV genotypes.

Number of coinfections	Number (*N*)	Relative Frequency (%)
2	46	79.3%
3	9	15.5%
4	3	5.2%
Total	58	100%

#### 3.3.1 Prevalence of various hr-HPV genotypes

Fourteen different hr-HPV genotypes were detected in this study. HPV-56 was the most frequently detected genotype in this study (13.7%, *n* = 29/211), followed closely by HPV-39 (12.3%, 26/211). HPV-16 had a prevalence of 1.9% (*n* = 4/211) in this study. HPV-18 (0.9%, *n* = 2/211) was the least common genotype detected in this study. The prevalence of all other hr-HPV genotypes is summarized in Table 5 below.

**Table 5:** Prevalence of various hr-HPV genotypes.

hr-HPV Genotype	Number (*N*)	Relative Frequency (*N*/211)(%) (95%CI)
16	4	1.9 (0.5–4.8)
18	2	0.9 (0.1–3.4)
31	25	11.8 (7.8–17.0)
33	16	7.6 (4.4–12.0)
35	15	7.1 (4.0–11.5)
39	26	12.3 (8.2–17.5)
45	20	9.5 (5.9–14.3)
51	13	6.2 (3.3–10.3)
52	8	3.8 (1.7–7.3)
56	29	13.7 (9.4–19.1)
58	20	9.5 (5.9–14.3)
59	6	2.8 (1.1–6.1)
66	16	7.6 (4.4–12.0)
68	11	5.2 (2.6–9.1)
Total	211	100

### 3.4 Risk factors for BV and hr-HPV infection

Age, vaginal douching and HIV infection were significantly associated with BV (cOR: 1.06, 95% CI = 1.06–1.09, *p* < 0.001), (cOR: 1.66, 95% CI = 1.01–2.72, *p* = 0.04) and (cOR: 3.33, 95% CI: 1.83–6.95, *p* < 0.001), respectively, and the following were not associated with BV: age at sexual debut, number of sexual partners, condom use, vaginal douching, history of smoking, history of alcohol consumption or parity. hr-HPV infection was significantly associated with the number of sexual partners only (cOR = 1.74, 95% CI = 1.04–2.92, *p* = 0.04). The crude odds ratios of all risk factors analyzed are summarized in Table 6 below. Multicollinearity diagnostics revealed no significant correlations among risk factors (VIF range: 1.005–1.029; tolerance range: 0.972–0.995).

**Table 6:** Association between BV or HPV, and several risk factors.

Risk Factor	BV		hr-HPV	
	cOR (95% CI)	*p*	cOR (95% CI)	*p*
Age	1.06 (1.04–1.09)	**<0**.**001**	0.98 (0.96–1.00)	0.09
Age at sexual debut	1.02 (0.92–1.13)	0.68	1.01 (0.92–1.11)	0.87
Number of sexual partners	0.76 (0.41–1.40)	0.37	1.74 (1.04–2.92)	**0**.**04**
Condom Use	1.42 (0.73–2.77)	0.30	1.26 (0.68–2.34)	0.46
Vaginal Douching	1.66 (1.01–2.72)	**0**.**04**	1.03 (0.62–1.70)	0.91
Smoking	0.23 (0.03–1.76)	0.12	1.02 (0.32–3.32)	0.97
Alcohol Consumption	1.24 (0.62–2.51)	0.54	1.31 (0.67–2.57)	0.43
Parity	0.95 (0.46–1.97)	0.89	0.77 (0.44–1.49)	0.44
HIV Infection	3.33 (1.83–6.95)	**<0**.**001**	1.19 (0.63–2.22)	0.59

Bold *p-*values highlight statistically significant associations.

### 3.5 BV and pooled hr-HPV cotesting result combinations

Approximately 10% (49/498) of the women had BV/hr-HPV coinfections. The remaining BV/hr-HPV combinations are summarized in Table 7 below. A significant association was observed between BV and pooled hr-HPV status (inclusive of HPV-16, -18, -31, -33, -35, -39, -45, -51, -52, -56, -58, -59, and -66, and -68 genotypes) both before and after adjustment for age, vaginal douching, the number of sexual partners, condom use, parity, the age of sexual debut and HIV status (cOR = 1.93, 95% CI: 1.25–2.98, *p* = 0.03 and aOR = 2.38, 95% CI: 1.48–3.81), *p* < 0.001, respectively.

**Table 7:** BV and hr-HPV cotesting result combinations.

Cotesting result	*N*	%	aOR (95% CI)	*p*
hr-HPV positive/BV positive	49	9.8	2.38 (1.48–3.81)	<0.001
hr-HPV positive/BV negative	91	18.3		
hr-HPV negative/BV positive	74	14.9		
hr-HPV negative/BV negative	284	57.0		

### 3.6 Associations between BV and specific hr-HPV genotypes

BV was significantly associated with the HPV-39 genotype infection both before and after adjustment (cOR: 2.79, 95% CI: 1.25–6.20, *p* = 0.009) and (aOR: 3.23, 95% CI: 1.37–7.64, *p* = 0.007), respectively. The HPV-52 genotype infection was also significantly associated with BV both before and after adjustment: (cOR: 5.25, 95% CI: 1.24–22.32, *p* = 0.01) and (aOR: 5.38, 95% CI: 1.17–24.74, *p* = 0.03) respectively. The associations between BV and other hr-HPV genotypes after adjustment are summarized in Table 8. Detailed data on the association between BV/specific hr-HPV genotypes, including the number of positive and negative cases, are available in Supplementary Appendix S1.

**Table 8:** Adjusted odds ratios between BV and hr-HPV genotypes.

HPV Genotype	aOR	95% CI	*p* value
16	3.12	0.39–25.28	0.29
18	2.15	0.18–25.54	0.54
31	1.81	0.72–4.56	0.21
33	1.95	0.62–6.11	0.25
35	2.48	0.82–7.53	0.11
**39**	**3**.**23**	**1.37**–**7.64**	**0**.**007**
45	0.47	0.10–2.12	0.33
51	2.47	0.72–8.47	0.15
**52**	**5**.**38**	**1.17**–**24.74**	**0**.**03**
56	1.71	0.73–4.00	0.22
58	0.67	0.20–2.27	0.52
59	0.71	0.07–6.98	0.77
66	1.36	0.43–4.29	0.60
68	2.53	0.69–9.32	0.16

Bold *p-*values highlight statistically significant associations.

### 3.7 Pap smear results

Most patients (96.2%, 479/498) had negative for intraepithelial lesion or malignancy (NILM) results. The remaining results are displayed in Table 9 below. Atrophy was the most reported non-neoplastic finding in this study (11.4%, 57/498). 45 (9.0%) of the Pap smears analyzed in this study had features suggestive of BV; of these, only 16 were confirmed as having BV via PCR, yielding a positive predictive value (PPV) of 36.6% for detecting BV from Pap smears.

**Table 9:** Pap smear findings.

Interpretation	*N*	Frequency (%)
Unsatisfactory	3	0.6
NILM	479	96.2
ASC-US	9	1.8
LSIL	4	0.8
HSIL	3	0.6
Non-Neoplastic Findings		
Shift in flora suggestive of BV	45	9.0
Fungal organisms consistent with *Candida* spp.	29	5.8
*Trichomonas Vaginalis*	2	0.4
Atrophy	57	11.4
Reactive cellular changes associated with Inflammation and typical repair	40	8.0

NILM, negative for intraepithelial lesion or malignancy; ASCUS, Atypical squamous cells of undetermined significance; LSIL, Low-grade squamous intraepithelial lesion; HSI, High-grade squamous intraepithelial lesion.

### 3.8 Pap smear cytology/pooled hr-HPV result combinations

The majority of patients (70.7%) had NILM/hr-HPV-negative results. The remaining combinations are summarized in Table 10 below. There was no statistically significant association between cytology and pooled hr-HPV status (*p* = 0.16).

**Table 10:** Pap smear cytology/pooled hr-HPV result combinations.

Pap smear/pooled hr-HPV cotesting results	N (%)	*p*
Unsatisfactory/hr-HPV negative	2 (0.4)	0.16
Unsatisfactory/hr-HPV positive	1 (0.2)	
NILM/hr-HPV negative	352 (70.7)	
NILM/hr-HPV positive	127 (25.5)	
ASCUS/hr-HPV negative	4 (0.8)	
ASCUS/hr-HPV positive	5 (1.0)	
LSIL/hr-HPV negative	1 (0.2)	
LSIL/hr-HPV positive	3 (0.6)	
HSIL/hr-HPV negative	0 (0.0)	
HSIL/hr-HPV positive	3 (0.6)	

### 3.9 Specific hr-HPV genotypes detected in various Pap smear cytology categories

HPV-31, -35, and -58 were each detected twice in abnormal Pap smear results, and the following hr-HPV genotypes were detected once: HPV-16, -39, -45, -51, -52, -56, -59, -66, and -68. Only HPV-16 and -68 were significantly associated with the detection of Pap smear abnormality. Table 11 below shows the detailed cross-tabulation of specific hr-HPV genotypes and Pap smear results.

**Table 11:** Specific hr-HPV genotypes and Pap smear results cross-tabulation.

hr-HPV genotype	Unsatisfactory		NILM		ASCUS		LSIL		HSIL		*p*
	N	P	N	P	N	P	N	P	N	P	
**16**	**3**	**0**	**476**	**3**	**8**	**1**	**4**	**0**	**3**	**0**	**0**.**046**
18	3	0	477	2	9	0	4	0	3	0	0.961
31	3	0	456	23	8	1	3	1	3	0	0.353
33	3	0	463	16	9	0	4	0	3	0	0.720
35	3	0	466	13	9	0	3	1	2	1	0.075
39	2	1	455	24	8	1	4	0	3	0	0.088
45	3	0	460	19	9	0	4	0	2	1	0.845
51	3	0	467	12	8	1	4	0	3	0	0.627
52	3	0	472	7	9	0	3	1	3	0	0.317
56	3	0	451	28	9	0	4	0	2	1	0.909
58	3	0	461	18	8	1	4	0	*2*	1	0.202
59	3	0	474	5	9	0	3	1	3	0	0.168
66	3	0	464	15	8	1	4	0	3	0	0.747
**68**	**3**	**0**	**469**	**10**	**9**	**0**	**3**	**1**	**3**	**0**	**0**.**001**

Bold *p-*values highlight statistically significant associations.

N, Negative for specific hr-HPV genotypes in the left-hand side column; P, Positive for specific hr-HPV genotypes in the left-hand side column.

## 4 Discussion

This study was a cross-sectional analysis of baseline data from an ongoing cohort study that aimed to investigate whether BV is associated with a higher risk of hr-HPV persistence in women. The broad finding of this study is the significant association between BV and pooled hr-HPV status (including HPV-16, -18, -31, -33, -35, -39, -45, -51, -52, -56, -58, -59, -66, and -68). A more specific perspective and scope of this study revealed a statistically significant association between BV and two hr-HPV genotypes (HPV-39 and HPV-52).

A significant association between BV and pooled hr-HPV infection has been reported in previous studies (5, 17, 20), but many of those findings are considered controversial because of the subjective methods used to detect BV in some of those studies. The utility of PCR in this study strengthens the argument that BV is significantly associated with cervical hr-HPV infection. This strongly underscores the need for thorough treatment and follow-up of patients presenting with incident BV to minimize the risk of hr-HPV infection. Having established an association between BV and cervical pooled hr-HPV infection, this study further investigated which hr-HPV genotypes were largely responsible for this association. This is a very under-explored area, and reliable data on this topic are lacking. In this study, BV was significantly associated with HPV-39 and HPV-52 infection. Although the *p*-values for both associations were less than 0.05, the narrower 95% confidence interval for HPV-39 makes it a more precise estimate of the association. The wider 95% confidence interval for HPV-52 demonstrates that the reported association lacks precision due to the small number of detected cases (*n* = 8). Additional research with more HPV-52 cases is therefore recommended to precisely determine the magnitude of the association between BV and HPV-52. However, this study is not the first to report a significant association between BV and HPV-52 infection. Lin et al. previously reported a similar association; however, the significance of their findings is limited by their use of the Amsel criteria to diagnose BV [12]. According to Bhujel et al.*,* the Amsel criterion has a poor sensitivity of 50% (21). This limitation was overcome in this study by using PCR, which is more empirical and objective. The superior sensitivity of PCR compared with Nugent or Amsel criteria means prevalence estimates based on the former will be higher, which introduces a comparability dilemma with previous studies based on the latter criteria. The strong association between HPV-39 and BV in this study is a novel finding that has great potential to inform policy regarding the management of BV in women with a focus on minimizing HPV-39 infection.

Lin et al. reported that the reasons for the association between BV and cervical hr-HPV infection remain under-investigated (12). However, several authors have postulated that the following factors are responsible for this association: the production of sialidases by anaerobes; the induction of mediators of inflammation, such as lipoteichoic acid, by anaerobes; and the destabilization of the epithelial cytoskeleton, resulting in exfoliation and necrosis of the epithelium and facilitating HPV infection (8, 10). Additionally, Borgdorff et al. reported that BV destabilizes the epithelial cytoskeleton, leading to exfoliation and necrosis of the epithelium, which facilitates HPV infection (13). However, this list is not exhaustive, and further research is needed to obtain additional information, particularly on the biological plausibility of BV associations with the HPV-39 and HPV-52 genotypes.

The finding of a significant association between BV and pooled hr-HPV status in this study underscores the importance of effectively treating incident BV to minimize hr-HPV infections. One way to do this is the use of probiotics. This was supported by McDermott, who reported that certain bacterial communities are effective at fighting hr-HPV infections (22). Lin *et al*. suggested that the elimination of anaerobes via antibiotics followed by the application of an *L. crispatus* vaginal probiotic to repopulate the flora could be the best course of action to manage women with BV to prevent hr-HPV acquisition or to enhance hr-HPV clearance in women (23). Additionally, McDermott reported that a probiotic (Papilocare) is already on the market in Europe and has been shown in a randomized clinical trial to enhance hr-HPV clearance (22). Embracing these vaginal creams in Africa, which is overburdened by both infections and cervical cancer, has the potential to contribute considerably to reducing mortality due to cervical cancer. The other critical issue raised by several authors is the high proportion of women who suffer from recurrent BV within six months of antibiotic treatment (24, 25). In view of this, policymakers in Africa should formulate a mandatory follow-up period to detect and treat recurrent BV, with an overall focus on minimizing hr-HPV infection, and thus reducing the cervical cancer burden on the continent.

Numerous epidemiological studies in Africa have reported hr-HPV prevalence rates ranging from 16% to 30% (26). In this study, the hr-HPV prevalence was 28.1%, this is consistent with previous studies conducted in the region. However, some studies in the region reported higher hr-HPV prevalence rates: Mbulawa et al. (South Africa, 66.2%) and Mitchell et al. (Uganda, 45%) (27, 28). This was attributed to the enrollment of predominantly HIV-infected women in those two studies. HIV compromises the body's immune system and interferes with the body's ability to clear HPV infections (27). However, the reported hr-HPV prevalence in this study may not be representative of the entire population, as it enrolled only women aged 30 or older. The limited age range compromises the external validity of the research findings, but it was necessary to prevent the detection of transient infections in young women. Additionally, women enrolled in this study are those who voluntarily came for cervical cancer screening. These volunteers may have risk profiles that differ systematically from the general population. This introduces selection bias, potentially significantly underestimating the observed associations. All these hr-HPV incidence rates in Africa are markedly higher than the 11% reported in the USA by Hirth (29). Hirth attributed the low HPV prevalence in the USA to HPV vaccination, which had reached modest levels by the time of publication (29).

HPV vaccination was introduced in Kenya in 2019; however, uptake remains low due to limited acceptance (30). According to Moucherand et al., the bottlenecks to HPV vaccination include poor awareness, social norms, and a lack of funds for community outreach (30). Cheechi et al. investigated the impact of HPV vaccination over a 10-year period in England and reported a significant decrease in hr-HPV prevalence from 15% in 2008, before the implementation of HPV vaccination, to <2% in 2023 (31). This is the way to go if a considerable reduction in hr-HPV and cervical cancer in Kenya is to be witnessed. The authors therefore recommend that policymakers enhance strategies to improve HPV vaccination awareness and uptake.

In our study, HPV-56 was the most frequently detected hr-HPV genotype. This finding was consistent with a previous study that determined the frequencies of hr-HPV genotypes in patients previously treated for cervical lesions via loop electrosurgical excision procedure (LEEP) (32). The next most frequently detected genotypes were HPV-39, -31, -45, and -58. HPV-16 and -18 accounted for 2.7% of all hr-HPV genotypes detected in this study. This finding is consistent with a study conducted at a family planning center in Kenya, where the sum of the prevalence rates for HPV-16 and -18 was reported to be less than 10% (33). However, it is important to note that although HPV-16 detection was infrequent in this study, it is one of only two hr-HPV genotypes, together with HPV-68, whose presence was significantly associated with abnormal Pap smear detection. In Kenya, three types of HPV vaccines have been approved and are currently in use: Cervarix (bivalent vaccine), which targets HPV-16 and -18; Gardasil (quadrivalent vaccine), which targets HPV-6, -11, -16 and -18; and Gardasil-9 (nanovalent vaccine), which targets HPV-6, -11, -16, -18, -31, -33, -45, -52 and -58 (34). The percentages of hr-HPV genotypes targeted by the HPV vaccines currently in use in Kenya were 2.7% (by Cervarix or Gardasil) and 52.1% (by Gardasil-9). Therefore, the Gurdasil-9 vaccine offers more protection to the Kenyan population and should be recommended ahead of the other two vaccines. However, the promotion of HPV vaccines should be concurrent with the promotion of cervical cancer screening, as the former is not effective in patients already infected by hr-HPV. In this study, Pap smear-based cervical screening yielded few abnormal results because the population included in this study was regularly screened for cervical cancer.

This study also has several limitations. First, the study was conducted at a single but the largest referral hospital in Kenya. Although the hospital has a catchment area across all counties in Kenya, the authors cannot claim with certainty that this sample was representative of Kenya, which compromises the generalizability of the study's findings to the general Kenyan population. Second, this baseline assessment used a cross-sectional design, in which data were collected simultaneously to determine both BV and hr-HPV status. This study design limits the study's ability to determine which infection was acquired first, and therefore, temporality could not be established.

## 5 Conclusion

BV was found to be significantly associated with pooled HPV status, HPV-39, and HPV-52 infection in Kenyan women. These findings underscore the importance of treating BV promptly, as doing so may help minimize hr-HPV infection.

## Data Availability

The raw data sets used and/or analyzed during the current study are available without reservation from the corresponding author on reasonable request.
