# Supplementary material for: Bacterial vaginosis and specific high-risk human papillomavirus genotype infection: a cross-sectional study in Nairobi, Kenya
Source: Front Glob Womens Health. 2026 Jun 18;7:1816885. doi: 10.3389/fgwh.2026.1816885 (PMC13323511; doi:10.3389/fgwh.2026.1816885)
Supplement: Supplementary file 1 [file supplementaryfile1.docx]

**Appendix 1: Detailed data on the ssociations between BV and specific hr-HPV genotypes**

| **HPV-X** | **HPV-X^Neg^/BV^Neg^** | **HPV-X^Pos^/BV^Neg^** | **HPV-X^Neg^/BV^Pos^** | **HPV-X^Pos^/BV^Pos^** | ***X^2^*** | **df** | **Crude Values** | | | **Adjusted Values** | | |
| --- | --- | --- | --- | --- | --- | --- | --- | --- | --- | --- | --- | --- |
|  |  |  |  |  |  |  | cOR | 95% CI | *p* | aOR | 95% CI | *p* |
| 16 | 373 | 2 | 121 | 2 | 1.39 | 1 | 3.08 | 0.43-22.12 | 0.24 | 3.12 | 0.39-25.28 | 0.29 |
| 18 | 374 | 1 | 122 | 1 | 0.12 | 1 | 1.53 | 0.14-17.00 | 0.73 | 2.15 | 0.18-25.54 | 0.54 |
| 31 | 358 | 17 | 115 | 8 | 0.75 | 1 | 1.47 | 0.62-3.48 | 0.39 | 1.81 | 0.72-4.56 | 0.21 |
| 33 | 364 | 11 | 118 | 5 | 0.38 | 1 | 1.40 | 0.48-4.12 | 0.54 | 1.95 | 0.62-6.11 | 0.25 |
| 35 | 366 | 9 | 117 | 6 | 1.95 | 1 | 2.09 | 0.73-5.98 | 0.16 | 2.48 | 0.82-7.53 | 0.11 |
| **39** | **361** | **14** | **111** | **12** | **6.79** | **1** | **2.79** | **1.25-6.20** | **0.009** | **3.23** | **1.37-7.64** | **0.007** |
| 45 | 357 | 18 | 121 | 2 | 2.42 | 1 | 0.33 | 0.08-1.43 | 0.12 | 0.47 | 0.10-2.12 | 0.33 |
| 51 | 368 | 7 | 117 | 6 | 3.30 | 1 | 2.70 | 0.89-8.18 | 0.07 | 2.47 | 0.72-8.47 | 0.15 |
| **52** | **372** | **3** | **118** | **5** | **6.25** | **1** | **5.25** | **1.24-22.32** | **0.01** | **5.38** | **1.17-24.74** | **0.03** |
| 56 | 356 | 19 | 113 | 10 | 1.59 | 1 | 1.66 | 0.75-3.67 | 0.21 | 1.71 | 0.73-4.00 | 0.22 |
| 58 | 359 | 16 | 119 | 4 | 0.25 | 1 | 0.75 | 0.25-2.30 | 0.62 | 0.67 | 0.20-2.27 | 0.52 |
| 59 | 370 | 5 | 122 | 1 | 0.21 | 1 | 0.61 | 0.70-5.24 | 0.65 | 0.71 | 0.07-6.98 | 0.77 |
| 66 | 364 | 11 | 118 | 5 | 0.38 | 1 | 1.40 | 0.48-4.12 | 0.54 | 1.36 | 0.43-4.29 | 1.36 |
| 68 | 369 | 7 | 118 | 4 | 1.90 | 1 | 2.23 | 0.69-7.15 | 0.17 | 2.53 | 0.69-9.32 | 0.16 |

Footnote: HPV-X: Specific high-risk HPV, *X^2^*: Chi-square value, df: degrees of freedom
